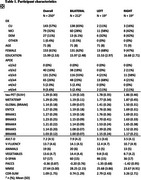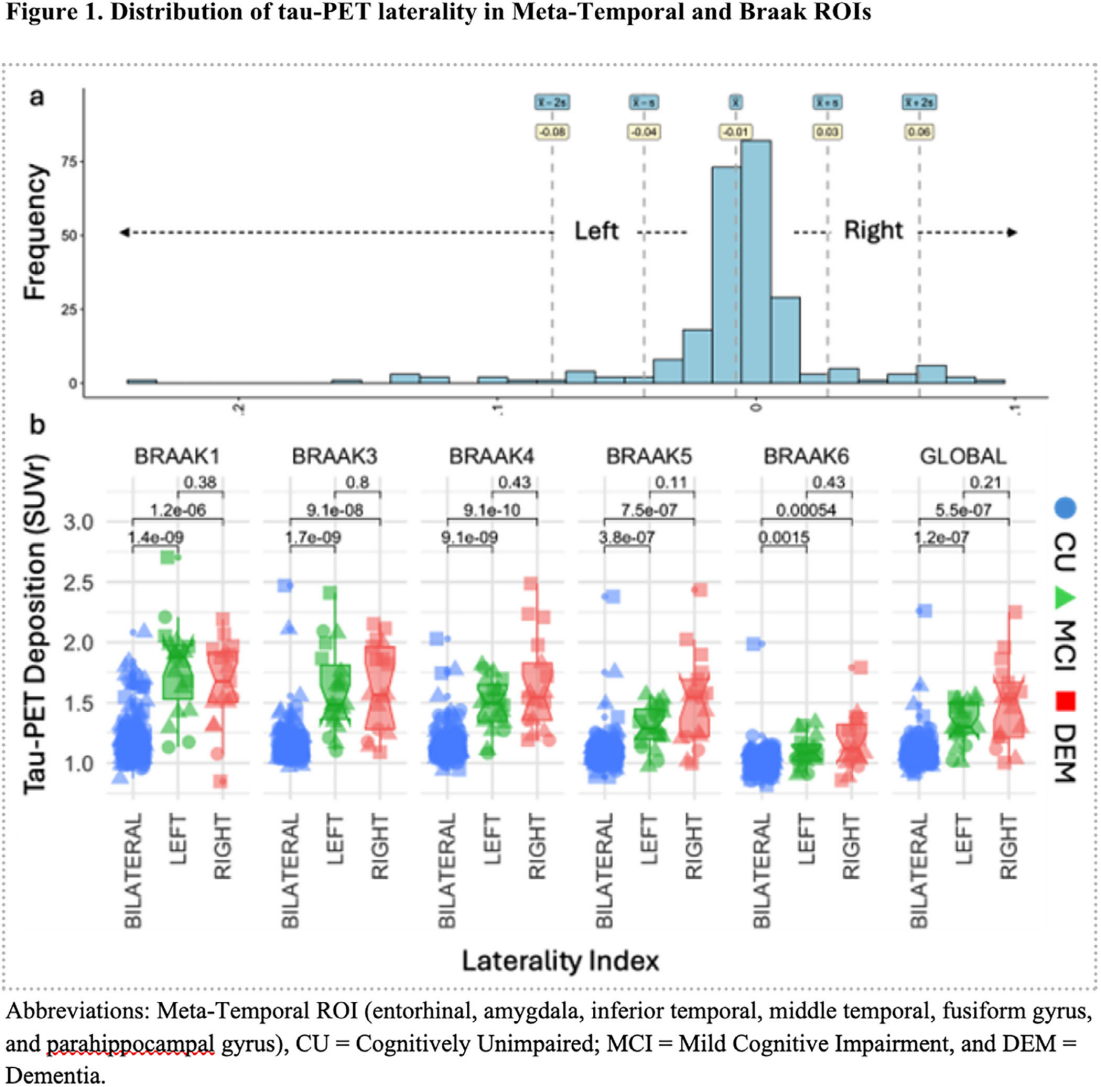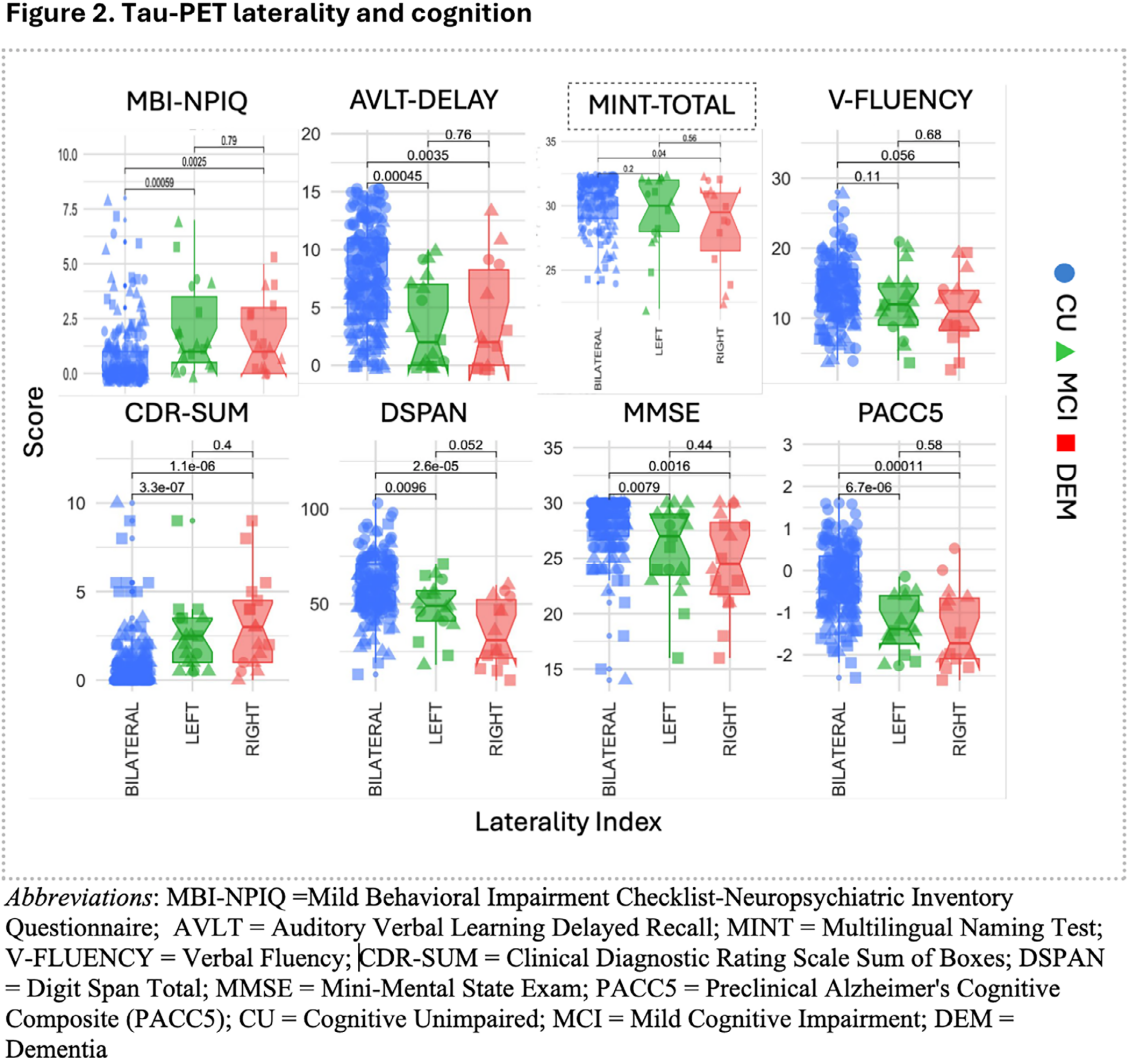# Evaluating tau‐PET deposition and laterality with cognition in the Wake Forest ADRC community‐based cohort

**DOI:** 10.1002/alz70856_105718

**Published:** 2026-01-09

**Authors:** Melissa M. Rundle, Da Ma, Kathryn H Alphin, Trey R. Bateman, Timothy M. Hughes, Kiran K. Solingapuram Sai, Suzanne Craft, Marc D. Rudolph

**Affiliations:** ^1^ Wake Forest School of Medicine, Winston‐Salem, NC, USA; ^2^ Wake Forest University School of Medicine, Winston‐Salem, NC, USA

## Abstract

**Background:**

Prior work suggests tau‐PET deposition patterns tracks strongly with cognitive deficits across general cognitive domains. Moreover, the degree to which tau‐PET deposition is left or right lateralized is associated with language versus behavioral impairment respectively.

**Method:**

Participants with tau‐PET data (*n* = 250; cognitively normal participants [CU; *n* = 143]; individuals with a consensus diagnosis of mild cognitive impairment [MCI; *n* = 79], dementia [DEM; *n* = 27], or otherwise not classified [OTHER; *n* = 1] enrolled in the Wake Forest ADRC (Table 1). Participants completed an annual battery of cognitive assessments including the Uniform Data Set (UDSv3, NACC), MMSE, CDR, a modified PACC5, NBI‐MPIQ, and MINT. Tau‐PET (SUVr) was quantified globally, regionally (entorhinal cortex, meta‐temporal), and according to BRAAK stage. Tau‐PET laterality indices were generated from these same regions. General linear models tested for associations between tau‐PET deposition or tau‐PET laterality with cognitive status and performance on UDSv3 assessments.

**Result:**

Overall, Tau‐PET deposition differed by cognitive status (CU < MCI < DEM; all *p* <.05) and was associated with poorer cognition across assessments evaluated (all *p* <.05; Table 1, Figures 1and 2). Associations were driven by tau‐PET+ participants irrespective of the threshold imposed or ROI used. Tau‐PET lateralization was detected in ∼23% of participants globally using a lenient criteria (e.g. SD^3^1; LEFT: 15%; RIGHT: 8%; Table 1), but did not differ by cognitive status (*p* = .17) and was not associated with cognition.

**Conclusion:**

Overall, increased Tau‐PET was associated with poorer cognition and differed by cognitive status as expected. Due to the small number of participants with detectable levels of laterality, we did not find any association between laterality and cognition. In future work we plan to quantity tau pet patterns at the voxel level and utilize a machine‐learning based staging scheme to improve characterization of AD progression and subtypes (e.g., SusTain), as well as take advantage of additional open‐source datasets with tau data.